# CD27^−^ B-Cells Produce Class Switched and Somatically Hyper-Mutated Antibodies during Chronic HIV-1 Infection

**DOI:** 10.1371/journal.pone.0005427

**Published:** 2009-05-01

**Authors:** Alberto Cagigi, Likun Du, Linh Vu Phuong Dang, Sven Grutzmeier, Ann Atlas, Francesca Chiodi, Qiang Pan-Hammarström, Anna Nilsson

**Affiliations:** 1 Department of Microbiology, Tumor and Cell biology, Karolinska Institutet, Stockholm, Sweden; 2 Department of Laboratory Medicine, Karolinska Institutet at Karolinska University Hospital Huddinge, Stockholm, Sweden; 3 Gay Men's Health Clinic, The South Hospital, Stockholm, Sweden; 4 Department of Medicine Solna, Infectious Diseases Unit, Karolinska University Hospital, Stockholm, Sweden; 5 Department of Women and Child Health, Karolinska Institutet, Stockholm, Sweden; New York University School of Medicine, United States of America

## Abstract

Class switch recombination and somatic hypermutation occur in mature B-cells in response to antigen stimulation. These processes are crucial for the generation of functional antibodies. During HIV-1 infection, loss of memory B-cells, together with an altered differentiation of naïve B-cells result in production of low quality antibodies, which may be due to impaired immunoglobulin affinity maturation. In the current study, we evaluated the effect of HIV-1 infection on class switch recombination and somatic hypermutation by studying the expression of activation-induced cytidine deaminase (AID) in peripheral B-cells from a cohort of chronically HIV-1 infected patients as compared to a group of healthy controls. In parallel, we also characterized the phenotype of B-cells and their ability to produce immunoglobulins *in vitro*. Cells from HIV-1 infected patients showed higher baseline levels of AID expression and increased IgA production measured *ex-vivo* and upon CD40 and TLR9 stimulation *in vitro*. Moreover, the percentage of CD27^−^IgA^+^ and CD27^−^IgG^+^ B-cells in blood was significantly increased in HIV-1 infected patients as compared to controls. Interestingly, our results showed a significantly increased number of somatic hypermutations in the VH genes in CD27^−^ cells from patients. Taken together, these results show that during HIV-1 infection, CD27^−^ B-cells can also produce class switched and somatically hypermutated antibodies. Our data add important information for the understanding of the mechanisms underlying the loss of specific antibody production observed during HIV-1 infection.

## Introduction

The ability of B-cells to differentiate into antibody secreting cells (ASC) that produce highly specific antibodies (Abs) is the key for a successful immune response against pathogens [1]. Upon encountering an antigen (Ag), mature naïve B-cells migrate to the secondary lymphoid organs where they organize germinal centers (GC) and undergo immunoglobulin (Ig) affinity maturation [2]. Two physiological processes are taking place at this stage, class switch recombination (CSR) and somatic hypermutation (SHM). CSR allows a previously rearranged Ig heavy chain variable (V) domain to be expressed in association with a different constant region, leading to production of different isotypes (IgG, IgA or IgE), with improved effector functions [3], [4], [5]. In SHM the V domains of immunoglobulin may increase their affinity by accumulations of mutations. These processes are fundamental for the quality of the immune response and for development of an efficient serologic memory to prevent re-infections [1].

During HIV-1 infection the B-cell compartment displays several alterations [6]; naïve B-cells, which normally lack CD27 expression [7], have an altered expression of several differentiation markers and hypergammaglobulinemia is frequently observed [8], [9]. However, despite high levels of serum IgG, the patients are unable to mount specific immune responses towards HIV-1 [10] or other pathogens [8]. This is partly due to a loss of memory B-cells (defined as CD19^+^CD27^+^) [7] which undergo spontaneous apoptosis [11] leading to loss of serologic memory [12]. Although the currently available Highly-Active Anti Retroviral Therapy (HAART) is able to normalize the proportion of different B-cell subpopulations in chronically HIV-1 infected patients [13], serologic memory cannot be restored [12]. This suggests that primary HIV-1 infection might be a crucial event for the elimination of Ag-specific subpopulations of memory B-cells [14].

CSR and SHM are both dependent on the expression of the enzyme activation-induced cytidine deaminase (AID) [3], [15]. Moreover, CSR is a highly regulated process, controlled by soluble cytokines and by T-cell membrane interaction between CD40 ligand (CD40L) and CD40 on the B-cell surface [3], [16]. In humans, switching to IgG is stimulated by the cytokines IL-10 and IL-4 whereas IgA expression is also promoted by TGF-β [17], [18]. The evidence that isotype switching is a highly CD40-dependent event is demonstrated in the hyper-IgM syndrome, where almost no secondary isotypes are produced as a result of a mutated CD40L [19].

Different effects of purified HIV-1 proteins on CSR have previously been reported [20], [21] where nef was shown to be taken up by B-cells and subsequently suppressed CSR by inducing IκBα and SOCS proteins, thereby blocking CD40 ligand and cytokine signalling via NF-κB and STAT proteins. These data suggest that HIV-1 may evade T-cell dependent IgA and IgG responses through interaction between nef and B-cells [21]. However, purified env was shown to upregulate AID expression, thus inducing CSR in a subset of B-cells expressing mannose C-type lectin receptors (MCLRs) [20]. This subset of B-cells required B-cell-activating factor (BAFF) for up-regulation of the MCLRs in order for Ab secretion to occur. These results indicate that env triggers IgA and IgG responses by activating MCLR expressing B-cells through a CD40-independent pathway, thus impairing protective T-cell-dependent Ab responses [20]. Taken together, these data [20], [21] suggest that different mechanisms underlie the impaired T-cell dependent- and independent- Ab responses during HIV-1 infection.

In the current study, we evaluated the effect of HIV-1 infection on CSR by studying a cohort of chronically HIV-1 infected patients, with low levels of HIV-1 viral load and treated with HAART, as compared with a group of healthy controls. In particular, we characterized the phenotype of B-cells, measured the levels of AID expression and Ig production upon stimulation of peripheral blood mononuclear cells (PBMCs) *in vitro*. Moreover, we measured the percentage of CD27^−^ cells amongst all IgA^+^ and IgG^+^ B-cells in blood and analyzed the VH region of Ig transcripts.

## Methods

### Study design and population

Blood from 38 HIV-1 infected individuals and 29 healthy controls were collected. In the HIV-1 cohort, the median CD4^+^ T-cell count was 752 cells/µl (range 70–1164 cells/µl) and the viral load ranged from <20 to 681 copies/ml blood. The viral load was determined using the NASBA system (Organon Teknika, Boxtel, the Netherlands). In order to minimize the impact of viral replication on the studied parameters, all the recruited patients were under HAART with a median time of 83 months of treatment (13–188 months). Due to the limited amount of blood available from a single individual, all patients could not be included in all analyses (23 controls and 22 patients for studies of AID expression and measurement of IgA and IgG levels, 20 controls and 20 patients for IgA intracellular staining, 17 controls and 25 patients for IgG intracellular staining and 4 controls and 4 patients for sequencing of the VH regions from different B-cell subpopulations). Written informed consent was obtained from all subjects before enrolment and the ethical committees of the Karolinska University Hospital and Karolinska Institutet approved the study.

### Cell preparation and flow cytometry

PBMC from the study subjects were purified from 20–30 mL of EDTA-treated whole blood by centrifugation over a Ficoll-Hypaque density gradient. Flow cytometric cell-phenotyping on fresh isolated PBMCs were performed using the following fluorochrome conjugated anti-human Abs: anti-IgA-fluorescein isothiocyanate (FITC) (Dako, Stockholm, Sweden), anti-IgG-FITC (Pharmingen, San Diego, CA), anti-CD27-phycoerythrin (PE) (Pharmingen, San Diego, CA) and anti CD19- Cy-Chrome (Cy5) (Pharmingen, San Diego, CA) in addition to isotype control Abs (Pharmingen, San Diego, CA) to set the background staining intensity. IgA and IgG were detected with intracellular staining by the IntraStain kit (Dako, Stockholm, Sweden). The analysis was performed with a FACScalibur instrument (Becton Dickinson, Mountain View, CA, USA) using the CellQuest software (Becton Dickinson, Mountain View, CA, USA). Forward/side scatter dot plot was used to gate the live lymphocyte population. Anti-IgD-FITC, anti CD27-PE and anti CD19-Cy5 (Pharmingen, San Diego, CA) were used for cell sorting on a FACSAria instrument (Becton Dickinson, Mountain View, CA, USA) using the FACSDiva software (Becton Dickinson, Mountain View, CA, USA).

### Activation of B-cells in PBMC cultures

PBMCs were washed in phosphate-buffered saline (PBS) and resuspended in complete RPMI medium (Sigma, Stockholm, Sweden). Cells were cultured in 48 well plates at a concentration of 1×10^6^ cells/well with 1 ml of medium or medium supplemented with 1 µg/ml of anti-CD40 mAb (Diaclone, Besancon, France), 100 ng/ml IL-4 (ImmunoTools GmbH, Germany) and 50 ng/ml IL-10 (Peprotech, London, UK) or 10 µg/mL cytosine guanine dinucleotide (CpG) oligodeoxynucleotide (ODN) 2006 (CpG type B) (Microsynth, Balgach, Switzerland) at 37°C in 5% CO_2_. The cultures were run in duplicates and after 48 hrs of culture, cells were collected for total RNA extraction while cells in the remaining wells were maintained in culture until supernatants were collected at day 9 and stored at −20°C until tested. The total number of B-cells was analyzed by FACS and differences between the samples were not statistically significant.

### Enzyme-Linked Immunosorbent Assay (ELISA)

Total IgA and IgG Abs were measured in 9-day culture supernatants. Briefly, 96 well plates (Corning Inc., NY, USA) were coated with 50 µl/well of goat anti-human IgA and G (H+L) (Dako, Stockholm, Sweden) diluted 1∶1000 in Sodium Carbonate buffer 0.1 M overnight at +4°C. Plates were then washed four times with PBS containing 0.1% Tween-20 (Sigma, Stockholm, Sweden) and samples with serial dilutions (experiments were performed in duplicates) in PBS-Tween were added to the plates and incubated overnight. Plates were then washed four times with PBS-Tween and Horseradish peroxidase (HRP)-conjugated rabbit anti-human IgA or IgG (Dako, Stockholm, Sweden) was added and the plates were subsequently incubated for 1 hour at room temperature. After a final wash, the plates were incubated with HRP substrate buffer until absorbances were read with a Labsystems Multiscan Bichromatic plate reader at a wavelength of 405 nm and analysed with a Deltasoft II programme (BioMetallics, Princeton, NJ, USA). The fold difference of IgA and IgG production between non-stimulated and stimulated cells was calculated by dividing the levels of Igs after stimulation with the spontaneous Igs production of non-stimulated cells left in culture medium for 9 days.

### RNA isolation and first-strand cDNA synthesis

Total RNA was purified using the RNeasy minikit (QIAGEN, Valencia, CA, USA) from purified PBMCs (approximately 1×10^6^ cells) or sorted B-cell subpopulations (2–8×10^5^ cells). Total RNA was reverse transcribed into first-strand cDNA using a using a cDNA synthesis kit (GE Healthcare, Stockholm, Sweden).

### Quantitative real time PCR

Quantitative real time polymerase chain reaction (RT-PCR) was performed on an ABI PRISM 7900HT sequence detection system (Applied Biosystems, Stockholm, Sweden). The primers used for amplification of the AID transcripts were the following: forward 5′-CACCACTATGGACAGCCTCTTG-3′ and reverse 5′-ACTGTCACGCCTC-TTCACTACG-3′, and for endogenous control β-actin: forward 5′-CTGGCACCACACCTTCTACAA-3′ and reverse 5′-CAGCCTGGATAGCAACGTACA-3′. Amplification was performed for 40 cycles, each cycle consisting of 94°C for 50 sec, 62°C for 1 min, and 72°C for 1 min. The relative AID expression level was calculated as described previously [22].

### PCR amplification and analysis of the VH-Cγ clones

The primers used for amplification of VH3-Cγ and VH3-23-Cγ transcripts were VH3-consensus (5′-aatctagaGGTGCAGCTGGTGGAGTC-3′) or VH3-23 (5′-tctagaGGCTGAGCTGGCTTTTTCTTGTGG-3′) and CγB (5′-cagtcgacAAGACC-GATGGGCCCTTGGTGG-3′). The oligonucleotides contained a restriction site (underlined, a Xba I site in the in the VH3-consensus or VH3-23 primer and a Sal I site in the CγB primer) for directional cloning of the PCR products. Amplification was performed in 35 cycles, each cycle consisting of 94°C 50 sec, 62°C 1 min and 72°C 1 min. High fidelity DNA polymerases Vent (New England BioLabs, Hertfordshire, England, GB) or Pfu (Fermentas Life Sciences, Burlington, Canada) were used for the amplification. The PCR products were purified and cloned into the Bluescript II KS (+) vector (Stratagene, La Jolla, CA, USA) and transformed into JM 109 competent cells. The resulting clones were screened by PCR amplification (VH3-consensus or VH3-23 and CγB) and positive clones were sequenced by an automated fluorescent sequencer in the MWG Co. (Macrogen, Seoul, South Korea). Sequence analysis was performed using the IMGT/V-QUEST (http://imgt.cines.fr) to align the VH-CγB sequences to their closest germline VH, D, and JH segment counterparts.

### Statistical analyses

Regression analyses were performed using Sigma Stat for Windows software (SPSS Inc, Chicago, USA). Differences between patients and controls were analysed by parametric (t test) or non-parametric (Mann-Whitney-U-test) tests. Results are presented as mean values±standard deviation or percentages.

## Results

### AID expression is different in controls and HIV-1 infected patients *ex-vivo* but it reaches similar levels following anti-CD40 and TLR9 stimulation *in-vitro*

**Figure 1:**
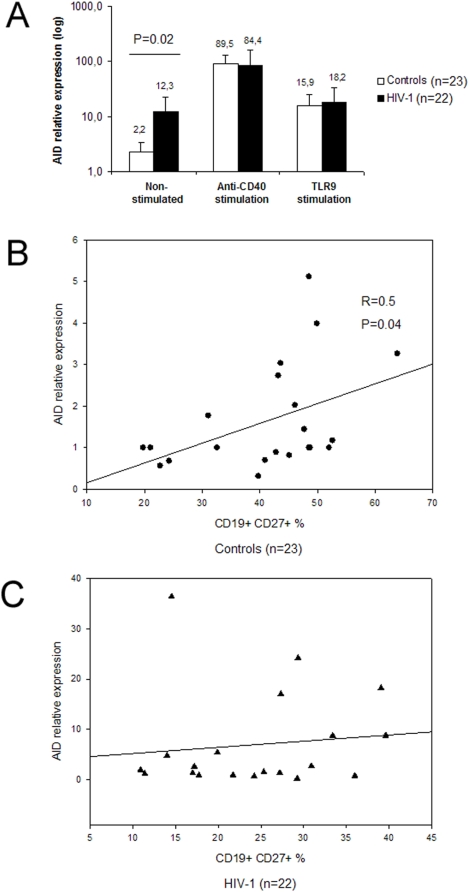
AID expression in HIV-1 infected patients and healthy controls. (A) The *ex-vivo* levels of AID mRNA expression (left panel) are higher in patients (black bars) as compared with controls (white bars) but they reach similar levels upon *in vitro* stimulation (middle and right panels). The baseline levels of AID mRNA expression correlate with the percentage of CD27^+^ B cells in healthy controls (B) while there is no correlation for HIV-1 infected patients (C). The anti-CD40 stimulation was performed with an anti-CD40 mAb, IL4 and IL10 while TLR9 stimulation with CpG.

The baseline level of AID mRNA expression in non-stimulated cells was higher in chronically HIV-1 infected patients (12.3±10.0) as compared to healthy controls (2.2±1.1) (P = 0.02) (Fig. 1A, left panel). Following 48 hours of anti-CD40 stimulation (anti-CD40 mAb, IL4 and IL10) AID expression reached similar levels in both patients (84.4±74.4) and controls (89.5±41.5) (Fig. 1A, middle panel). Following 48 hours of TLR9 stimulation, AID expression also reached similar levels in both patients (18.2±14.1) and controls (15.9±8.5) (Fig. 1A, right panel). In order to test whether AID expression correlated to the proportion of CD27^+^ memory (CD27^+^) B-cells, we compared the AID expression at baseline and the CD27^+^ B-cells percentage by regression analyses. Interestingly, a statistically significant correlation (R = 0.5, P = 0.04) was observed in the control group (Fig. 1B) but not in patients (Fig. 1C). The expression of AID was subsequently investigated in sorted CD27^−^ and CD27^+^ B-cells from a subgroup of patients (n = 3) and controls (n = 3). Although, due to the small number of samples tested, the difference did not reach statistical significance, a higher level of AID expression in patient CD27^−^ B-cells was observed (40.3±33.1 vs 12.6±2.7 in controls), whereas a lower level of AID expression was observed in the CD27^+^ compartment for patients (9.7±8.0 vs 64.0±37.0 in controls).

### Blood cells from HIV-1 infected patients produce higher levels of IgA *in vitro*

**Figure 2:**
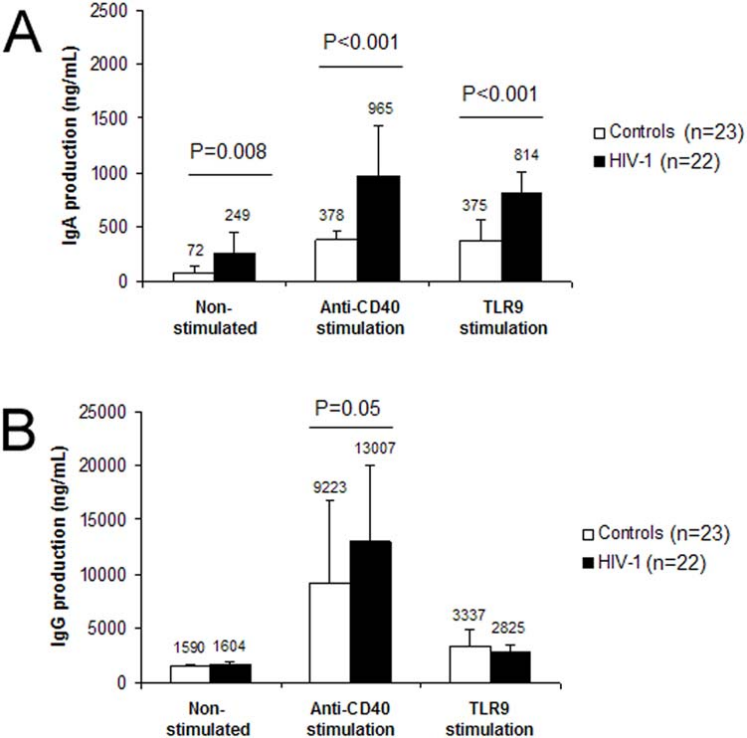
IgA and IgG production *in vitro* in HIV-1 infected patients and healthy controls. (A) The levels of IgA before (left panel) and after *in vitro* stimulation (anti-CD40 mAb, IL4 and IL10 or CpG, middle and right panels) are higher in patients (black bars) as compared with controls (white bars). (B) The levels of IgG before (left panel) and after *in vitro* stimulation (middle and right panels) are similar in patients (black bars) as compared with controls (white bars).

To assess Ig production from B-cells, PBMCs isolated from the blood of HIV-1 infected patients were cultured *in vitro*. High levels of IgA were spontaneously produced in the culture medium by patient B-cells (249±193 ng/mL) as compared to healthy controls (72±71 ng/mL) (P = 0.008) (Fig. 2A, left panel). The same trend was found after 9 days of stimulation with anti-CD40 Ab (965±468 vs 378±77 ng/mL) (P<0.001) (Fig. 2A, middle panel) or TLR9 stimulation with CpG (814±189 vs 375±191 ng/mL) (P<0.001) (Fig. 2A, right panel). No differences between patients and controls were however observed for spontaneous IgG production (1604±296 vs 1590±128 ng/mL) (Fig. 2B, left panel) while upon anti-CD40 stimulation, IgG production reached slightly higher levels in patients (13007±7005 ng/mL) as compared to controls (9223±7590 ng/mL) (P = 0.05) (Fig. 2B, middle panel). The TLR9 stimulation is less potent as compared to anti-CD40 stimulation but induced similar IgG levels in patients (2825±594 ng/mL) and controls (3337±1468 ng/mL) (Fig. 2B, right panel). Interestingly, stimulation through TLR9 induced a low fold increase of IgG in both the patient and control groups (1.8 vs 2.1 fold) as compared with anti-CD40 stimulation (8.1 vs 5.8 fold).

### Expanded populations of CD27^−^ IgA^+^ and CD27^−^ IgG^+^ B-cells are found in the blood of HIV-1 infected patients

**Figure 3:**
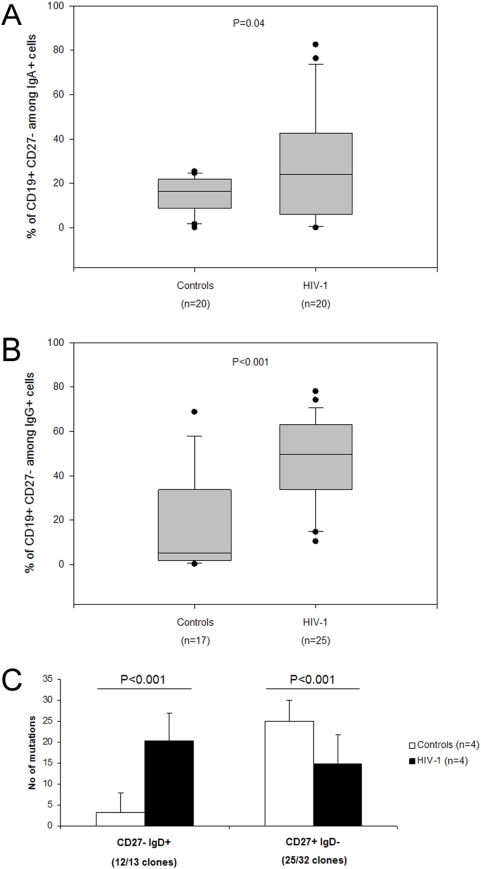
HIV-1 infected patients present with expanded populations of blood CD27^−^ IgA^+^ and CD27^−^ IgG^+^ B-cells and show inverse patterns of SHM. The percentage of CD27^−^IgA^+^ (A) and CD27^−^IgG^+^ (B) among total IgA/G expressing B-cells is significantly expanded in HIV-1 infected patients (right panel) as compared with healthy controls (left panel). (C) The number of somatic hypermutations in the VH region of mRNA transcripts of CD27^−^ B cells (left panel) is increased in HIV-1 infected patients (black bars), as compared with healthy controls (white bars), while an opposite trend is shown for CD27^+^ B cells (right panel), where the number of somatic hypermutations in the VH region of mRNA transcripts is decreased in HIV-1 infected patients (black bars) as compared with healthy controls (white bars). The number below the bars indicates the number of clones analysed.

The high baseline levels of AID together with the finding that AID expression did not correlate with the CD27^+^ B-cell counts in HIV-1 infected patients might suggest the involvement of CD27^−^ B-cells in Ig production. In order to investigate whether CD27^−^ B-cells in HIV-1 infected patients produce class switched Abs, we measured the percentage of CD27^−^ B-cells amongst IgA^+^ or IgG^+^ B-cells in the blood of patients and controls. Intriguingly, the results showed a significant increase of the percentage of CD27^−^ B-cells among all intracellular IgA (27 vs 15%, P = 0.04) and IgG (47 vs 19%, P<0.001) positive cells in patients as compared to healthy controls (Fig. 3A and B).

### The VH genes in CD27^−^ B-cells from HIV-1 infected individuals are highly mutated

In order to evaluate the ability of different B-cell sub-populations to produce somatically hypermutated Abs, we sorted cells from 4 additional patients and 4 healthy individuals by flow cytometry. To increase the purity of the sorting, CD19^+^CD27^−^ and CD19^+^CD27^+^ B-cells were also sorted according to the surface expression of IgD. The VH region of the mRNA transcripts for CD19^+^CD27^−^IgD^+^ and CD19^+^CD27^+^IgD^−^ B-cells were PCR amplified using either a VH3 consensus primer or a VH3-23 specific primer, together with a Cγ specific primer. In total, 53 and 49 VH-Cγ sequences were generated from B-cells of patients and controls respectively (Table 1). Among those, 45 and 37 represented distinct B-cell clones, with unique complementarity-determining region 3 (CDR3) sequences and these clones were included in the subsequent SHM pattern analysis. As shown in Table 1 and Figure 3C, while the VH genes amplified from CD19^+^CD27^−^IgD^+^ cells in the control group had a low number of mutations, as expected (average 3 mutations per gene; mutated in 1.2% bp sequenced), the VH genes from patient CD19^+^CD27^−^IgD^+^ cells had a significantly higher number of mutations (20 mutations per gene, P<0.001; mutated in 6.3% bp sequenced). Conversely, the VH genes from the CD19^+^CD27^+^IgD^−^ cells from patients, which would be expected to have a higher number of mutations, had a significantly reduced number of mutations as compared to the controls (15 vs 25 mutations per gene respectively, P<0.001; 4.8% vs. 8.3% bp sequenced). The ratio of replacement vs silent mutations (R/S) was similar in the patient and control groups for both CD27^+^ and CD27^−^ cells, suggesting that there was no major difference in the Ab selection process (Table 1).

**Table 1:** Mutations in VH3-Cγ transcripts from sorted CD27−IgD+ and CD27+IgD− B cells from HIV-1 infected patients and healthy controls.

	All clones	Distinct	Unmutated	Total mutations in V region	Total bp sequenced	Frequency of mutations (% bp)	*R/S **CDRs-**FRs
HIV-1							
CD27−IgD+	20	13	0	240	3809	6.3	6.1–2.1
CD27+IgD−	33	32	0	443	9178	4.8	4.2–2.1
Controls							
CD27−IgD+	20	12	9	40	3406	1.2	6.6–1.0
CD27+IgD−	29	25	0	592	7105	8.3	4.6–2.1

The SHM pattern analysis was performed in the distinct clones.

* Replacements/silent mutations.

** Complementarity-determining region.

*** Framework region.

## Discussion

During primary HIV-1 infection, a high degree of viral replication and CD4^+^ T-cell depletion occur. In parallel, a large proportion of the CD27^+^ B-cells die by apoptosis while CD27^−^ B-cells are characterized by a modified phenotype [14]. Successful HAART normalizes B-cell subpopulation percentages in blood [13]. During HAART, however, many commensal and opportunistic micro-organisms which are normally kept under control by the immune system, are more active within the respiratory tract [23], the gut [24] and the brain [25] resulting in the presence of microbial products in the body [26]. In this scenario, B-cells may be more susceptible to activation and differentiation into ASC by polyclonal stimuli [27]. During chronic HIV-1 infection, Ag specific Ab titres are decreased while the total amount of circulating IgG is elevated (hypergammaglobulinemia) [10].

We report a higher baseline AID expression in B-cells from patients with chronic HIV-1 infection as compared to healthy controls. Moreover, the levels of AID at baseline did not correlate to the proportion of the CD27^+^ B-cells in the patients suggesting that CD27^−^ B-cells may contribute to an increased baseline AID expression. AID up-regulation is normally associated with GC B-cells [28]. However, high levels of extra-follicular AID expression have previously been reported in non-stimulated PBMCs from HIV-1 infected patients and found to be associated with development of AIDS associated non-Hodgkin B-cell lymphoma (AIDS-NHL) [29]. In addition, it has been shown in a mouse model that over expression of AID in B-cells induced production of pathogenic multireactive Abs [30]. In HIV-1 infection, several studies have reported the presence of multi-reactive low affinity Abs in serum and it is possible that high baseline AID expression leads to the production of auto Abs.

Bacterial CpG DNA motifs have previously been shown to activate B-cells by TLR9 ligation which induces IgG CSR [31]. Recently, a viral double stranded RNA was shown to induce production of class switched Abs through the engagement of TLR3 in the upper respiratory tract mucosa [32]. We found that in B-cells from patients with chronic HIV-1 infection, AID expression was induced *in vitro* upon CD40-dependent and TLR9 stimulation but less efficiently than in controls where the fold increase was much higher. Interestingly, it has been shown that B-cells from aged mice respond poorly to anti-CD40 activation and have a lower AID expression compared to B-cells from young mice [33]. Our finding may therefore support the hypothesis that B-cells are prematurely exhausted during HIV-1 infection [34]. On the other hand, despite the higher baseline AID expression observed in B-cells from patients, the levels of AID reached similar levels in stimulated cells from both patients and controls. The lower fold increase observed in patients could also be interpreted as a result of an inefficient stimulation in the patient samples as they are already partly stimulated. In our experiments, the consequences of virus particles, present during primary infection and non-treated chronic infection, were minimized since all the patients received HAART. Thus, the ability of B-cells during chronic infection to express AID, the prerequisite for CSR, in response to different stimuli could be evaluated *in vitro*. However, the presence of a small fraction of T-cells carrying replication competent HIV-1 virus in our cultures [35] cannot be excluded.

We also investigated Ig production *ex vivo* and upon anti-CD40 and TLR9 stimulation. High IgA levels were found in cultures from non-stimulated HIV-1 patient cells as well as upon anti-CD40 and TLR9 stimulation where patient cells secreted more IgA as compared to controls. In this cohort of treated patients, we could not detect any significant increase in spontaneous IgG secretion. This is in accordance with a previous study where the majority of aviremic HAART treated patients had normal IgG levels [36]. Moreover, we observed that anti-CD40 stimulation only significantly increased IgG but not IgA production in both patients and controls. This might be due to the combined effect of the anti-CD40 Ab and of the cytokines IL-10 and IL-4 which are known to induce switching towards IgG, whereas IgA expression is also promoted by TGF-β [17], [18]. On the other hand, TLR9-driven activation of B-cells induced a high IgA production in patient cells as compared to controls. This might be a common and physiological phenomenon in the gut where the majority of Abs produced against commensal bacteria are of the IgA class [37], [38]. Thus, the increased ability of B-cells from HIV-1 infected patients to respond to stimulation and produce IgA, might reflect an increased response to microbial products [26] in HIV-1 infected individuals. Interestingly, the levels of IgA and IgG produced spontaneously and upon stimulation in culture were higher in cultures with cells from patients with CD4 <350 cells/µl of blood compared to patients with higher CD4 T-cell counts, although the results did not reach statistical significance (data not shown).

Our results on AID expression and the lack of correlation to the memory B-cell population in HIV-1 infected individuals may suggest that CD27^−^ B-cells participate in the production of class switched Abs during HIV-1 infection. This hypothesis is strongly supported by our additional findings that both CD27^−^IgA^+^ and CD27^−^IgG^+^ B-cells are expanded in the blood of HIV-1 infected patients. However, it is also possible that IgA^+^ or IgG^+^ memory B-cells from HIV-1 patients down-regulate CD27. In a previous report, metalloprotease inhibitors were shown to block the release of CD27 and enhance the immune stimulatory activity of chronic lymphocytic leukemia cells [39]. Ongoing HAART regimens consist of a cocktail of drugs often including a protease inhibitor; therefore it is possible that different HAART schedules may result in cleavage of the CD27 molecule from the cell surface [40]. The levels of soluble CD27 in HAART treated HIV-1 infected patients have previously been suggested as a marker of immune activation [41]. CD27^−^ B-cells expressing intracellular IgG have previously also been found to be expanded in patients suffering with systemic lupus erythematosus (SLE) [42]. In healthy individuals, CD27^−^ B-cells have also been described and defined as a new B-cell memory subtype expressing VH genes with low frequency of SHM [43]. To our knowledge, this is the first report showing the presence of IgA^+^ B-cells lacking the expression of CD27 in the blood of healthy individuals. However, while comparing the percentage of CD27^−^IgA^+^ and CD27^−^IgG^+^ B-cells to the AID expression and Ig production *ex-vivo* and upon *in vitro* stimulation, it was not possible to find a significant correlation between these parameters (data not shown). This could be due to the low percentages of these B-cell populations compared to others in peripheral blood.

In order to verify whether CD27^−^ B-cells represent a population of memory B-cells, we studied the SHM pattern in the Ig VH region of Cγ transcripts of CD27^−^IgD^+^ B-cells. Our data show that CD27^−^ B-cells sorted from HIV-1 infected patients contain Cγ transcripts in which the VH region carry a high number of somatic hypermutations compared to the controls. Therefore, the CD27^−^ B-cells in HIV-1 infected individuals may represent a part of the memory B-cell pool. Based on these data, we suggest that the CD19^+^CD27^−^IgD^+^ population of both controls and HIV-1 patients may contain B-cells that are in the process of switching to IgG/IgA. In this respect, it is possible that the co-expression of IgD and IgG/IgA by CD27^−^ B cells during HIV-1 infection is increased.

In addition, from the analysis on the Ig VH region from the sorted CD27^+^IgD^−^ B-cells, we could observe a decrease in the number of somatic hypermutations in HIV-1 patients compared to healthy controls. SHM occurs during B-cell development, in the GC reaction, and, upon Ag-encounter a new mutation may be inserted when B-cells replicate and divide. Our data suggests that CD27^+^ B-cells during HIV-1 are either depleted after a few cell divisions, or prevented from entering the GC. We have recently shown that during chronic HIV-1 infection, CD27^+^ and CD27^−^ B-cells show an altered migration patterns due to alterations in expression of the receptor-ligand pair CXCR5/CXCL13 [44]. The dysfunctional memory B-cell compartment in HIV-1 infected patients may thus be due to both altered CD27^−^ B-cells with a memory phenotype and exhausted CD27^+^ B-cells with impaired regulation of CSR. Evidence for HIV-associated B-cell exhaustion and dysfunctional memory B-cell compartment has recently been reported for HIV-1 infected viremic individuals [34]. Most importantly, clinical observations indicate that the loss of serological memory persists during chronic infection and that specific Ab titers cannot be recovered in patients who still present with hypergammaglobulinemia upon HAART treatment [8], [12].

We thus propose a model where CD27^−^ B-cells are more prone to respond to unspecific signals such as polyclonal bystander activation and TLR triggering by microbial products during HIV-1 infection. This would result in the expansion of CD27^−^ IgA^+^ and IgG^+^ B-cells [43] in blood. The levels of AID and the presence of a high number of somatic hypermutations in the Ig VH region of CD27^−^ B-cell transcripts demonstrated that these B-cell subpopulations have undergone Ig affinity maturation. In this scenario, the lack of specific immune responses during HIV-1 infection would be due both to the involvement of CD27^−^ B-cells in CSR and SHM, triggered by unspecific stimuli [31], [45], and to CD27^+^ B-cell exhaustion. This could be a consequence of persistent pathogen reactivation and microbial translocation shown during chronic HIV-1 infection [26]. A possible impairment in the TLR pathways in HIV-1 infected individuals should also be considered in the pathogenesis of the autoimmune phenomena occurring during HIV-1 infection [46] since there is increasing evidence that TLRs, reactive with autologous ligands, play a major role in these events [47].
